# Prevalence, Risk Factors, and Prognostic Factors of Primary Malignant Bone Neoplasms with Bone Metastasis at Initial Diagnosis: A Population-Based Study

**DOI:** 10.1155/2022/9935439

**Published:** 2022-03-26

**Authors:** Zheng-Wei Xiao, Hui-ling Guo, Hong-chao Chen, Lai-peng Yan, Yi-lin Liao, Shu-lin Li, Li-lan Zhao, Ling-bo Su, Jun-jie Li, Fa-qiang Tang

**Affiliations:** ^1^Shengli Clinical Medical College of Fujian Medical University, Fuzhou, Fujian Province, China; ^2^Department of Orthopaedics, Fujian Provincial Hospital, Fuzhou, Fujian Province, China; ^3^Department of Thoracic Surgery, Fujian Provincial Hospital, Fuzhou, Fujian Province, China

## Abstract

**Background:**

Bone metastasis (BM) has been proven to be responsible for the poor prognosis of primary malignant bone neoplasms (PMBNs). We aimed to identify the prevalence, risk factors, and prognostic factors for PMBNs patients with BM based on the Surveillance, Epidemiology, and End Results (SEER) database.

**Methods:**

4,758 patients diagnosed with PMBNs from 2010 to 2018 were selected from the SEER database. All patients were divided into two groups: the BM group or the non-BM group. Pearson's chi-square test and Fisher's exact method were used to assess baseline characteristics, and logistic regression analysis was applied to assess risk factors. In addition, a nomogram was constructed based on the results of Cox regression analysis among 227 patients with BM. The good performance and clinical applicability of the nomogram were tested by the concordance index, operating characteristic curve, area under the curve, calibration curves, and decision curve analysis.

**Results:**

227 (4.8%) patients had metastasis to bone at diagnosis. Primary site outside the extremities (axial: odds ratio, OR = 1.770; others: OR = 1.951), Ewing sarcoma (OR = 2.845), larger tumor size (5–8 cm: OR = 3.403; >8 cm: OR = 5.562), tumor extension beyond the periosteum (OR = 2.477), and regional lymph node metastasis (OR = 2.900) were associated with a higher risk of BM at the initial diagnosis of PMBNs. Five independent prognostic factors were found in the survival analysis: pathological type (chondrosarcoma vs. osteosarcoma: hazard ratio, HR = 0.342; Ewing sarcoma vs. osteosarcoma: HR = 0.592; and chordoma vs. osteosarcoma: HR = 0.015), marital status (HR = 2.457), pulmonary metastasis (HR = 1.934), surgery at the primary site (HR = 0.164), and chemotherapy (HR = 0.084). A nomogram based on these prognostic factors could be a good predictor of cancer-specific survival.

**Conclusions:**

We identified the prevalence, risk factors, and prognostic factors correlated with BM in PMBNs patients. The related nomogram could be a practical tool for therapeutic decision-making and individual counseling.

## 1. Introduction

Primary malignant bone neoplasms (PMBNs), which account for 0.2%-1% of human neoplasms, are mesenchymal tumors with a wide range of morphological and biological behaviors. [1] PMBNs have a tremendous impact on patients' life expectancy and quality of life, and osteosarcoma, Ewing sarcoma, chondrosarcoma, and chordoma are the four most common types [2]. Generally, patients present with pain and a mass at the primary site when PMBNs are first diagnosed. Radiographs usually show soft tissue masses and neoplasms with mixed osteoblastic and osteolytic destruction. [3] Although osteosarcoma, the most common type of PMBNs, is reported to occur in 0.2 to 3 per 100,000 people per year in the population, it is still one of the deadliest cancers during puberty [4].

Previous studies have shown that the mean survival times of patients with osteosarcoma, chondrosarcoma, Ewing sarcoma, and chordoma are approximately 54.9, 63.5, 58.1, and 66.9 months, respectively [5]. Metastasis was found to be a significant cause of worse prognosis and high mortality in these patients [6–8]. Metastatic dissemination in primary osseous neoplasms usually occurs hematogenously, with the lungs and bones being the most common metastatic sites. [9] A study based on 4,487 patients with detailed stage records found that 900 (19%) patients with primary malignant bone tumors had distant metastases at initial diagnosis, with lung metastases and bone metastases occurring in 11.2% and 4.6% patients, respectively [5].

Lung metastasis in bone malignancies has been defined as stage M1a by the American Joint Committee on Cancer (AJCC), and many studies have identified risk factors and prognostic indicators [10–12]. Metastasis to the bone, defined as stage M1b by the AJCC, has a worse prognosis than metastasis to the lung, and this conclusion has been confirmed by several studies [10, 13–16]. However, most of the studies on bone metastasis (BM) were based on small cohorts from single-center studies due to the rarity of the disease, which limits the application of the results. Due to the high mortality of PMBNs patients with BM, it is meaningful to investigate the related epidemiological features, risk factors, and prognostic indicators.

Nomograms are a frequently used feasible tool to predict the prognosis of diseases and have an edge over traditional predictive systems [17, 18]. Considered new standards for tumor evaluation, several nomograms have been constructed for bone tumors in recent years to accurately predict outcomes and provide precise guidance for clinical treatment [19–22]. However, no nomogram has been established for PMBNs patients with BM.

In the era of precision treatment with individualized medicine, we aimed to carry out a population-based analysis of this rare subgroup to identify the characteristics and various risk factors for PMBNs patients with BM. Then, we performed a survival analysis and developed a predictive nomogram to assist clinicians in establishing appropriate individualized treatment.

## 2. Materials and Methods

### 2.1. Study Population

**Figure 1:**
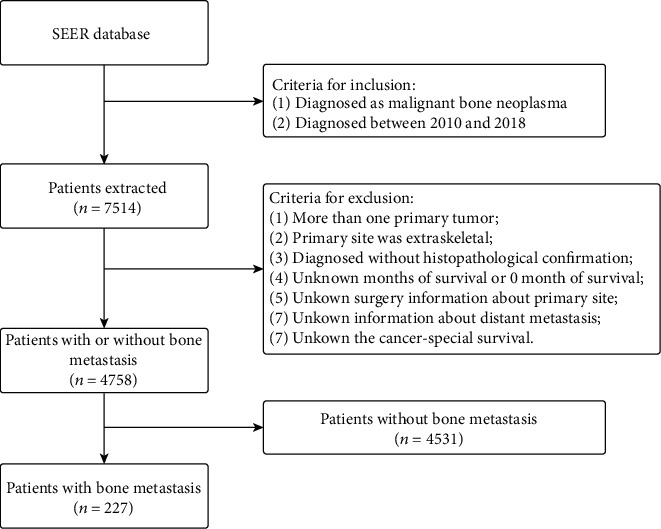
The flow chart of selection.

We conducted a retrospective study, and all patient data were obtained from the Surveillance Epidemiology and End Results (SEER) database, the most comprehensive source of cancer information, by using the SEER∗Stat application (version 8.3.9). The clinical information of patients with various cancers in the SEER database was collected from 18 population-based cancer registries, representing approximately 28% of the total population of the United States [23, 24]. The four most common types of malignant bone neoplasms were searched by using the International Classification of Diseases for Oncology (ICD-O-3) histological subtype codes. The time period was restricted to after 2010 since the information on distant metastases was incomplete before that. The final inclusion criteria for the study population were as follows: (I) patients with the four most common types of malignant bone sarcomas, including osteosarcoma (ICD-O codes: 9180, 9181, 9182, 9183, 9184, 9185, 9186, 9187, 9192, 9193, and 9194), chondrosarcoma (ICD-O codes: 9220, 9221, 9230, 9231, 9240, 9242, and 9243), Ewing sarcoma (ICD-O: 9260), and chordoma (ICD-O codes: 9370, 9371, and 9372); and (II) patients with malignant bone neoplasms diagnosed between 2010 and 2018. Patients were excluded according to the following criteria: (I) the presence of more than one primary tumor; (II) the primary site of tumors was extraskeletal; (III) the survival time was missing or the survival time code was 0 months; (IV) surgery information for the primary tumor was unavailable; (V) information about metastatic sites was unavailable; (VI) patients were diagnosed without histopathological confirmation; and (VII) cancer-special survival data were unavailable. Ultimately, a total of 4,758 patients were included.  Figure 1 shows the details of the inclusion and exclusion processes.

### 2.2. Variable Definition

The patient demographics of interest included age (<18 years old, 18 to 65 years old or >65 years old), sex (female or male), race (white, black, or other: American Indian/AK Native, Asian/Pacific Islander), and marital status (unmarried or married) at diagnosis. Clinical characteristics included tumor site (extremities: long and short bones of the upper and lower extremities; axial: pelvis, sacrum, coccyx, vertebral columns, sternum, clavicle, and ribs; or others: mandible, skull, and other atypical locations), tumor type (osteosarcoma, Ewing sarcoma, chondrosarcoma, or chordoma), tumor grade (low grade or high grade), tumor size (≤5 cm, 5-8 cm, or>8 cm), tumor extension (inside the periosteum or beyond the periosteum), and the presence or absence of regional node involvement, bone metastases, brain metastasis, liver metastasis, and lung metastasis. Treatment information included primary site surgery (yes or no), distant metastatic site surgery (yes or no), radiotherapy (yes or no/unknown), chemotherapy (yes or no/unknown), and the sequence of systemic therapy and surgery (no, before surgery, after surgery, or other).

When processing the information about tumor grade, we reclassified grade I and grade II (well-differentiated and differentiated) as low grade, whereas we reclassified grade III and grade IV (poorly differentiated and undifferentiated) as high grade, according to a previous report [25]. All data were grouped by combining the information provided by the SEER database with the experience of previous studies [26–29].

### 2.3. Outcome Measures

The primary outcome of our research was cancer-specific survival (CSS), which was defined as the survival span from first diagnosis to death due to primary bone sarcoma and was used to indicate the survival condition of patients.

### 2.4. Missing Data

Some data about race, marital status, tumor grade, tumor size, tumor extension, and metastases to regional lymph nodes were missing. Therefore, missing data was classified as ‘unknown' for statistical analysis.

### 2.5. Statistical Analysis

Categorical variables, presented as frequencies and percentages, were analyzed by using Pearson's chi-square test or Fisher's exact method to reveal the differences between groups with or without BM. CSS was calculated by the Kaplan–Meier (KM) method. Log-rank tests were performed to evaluate the potential differences in CSS between groups.

Demographic and clinical characteristics were analyzed by univariate and multivariate logistic regression to identify risk factors for PMBNs patients with BM. Variables with *P* < 0.05 in the univariate logistic regression analysis were analyzed by multivariate logistic analysis. The relevance between clinical characteristics and BM development was revealed by the odds ratios (ORs) and corresponding 95% confidence intervals (CIs).

Furthermore, to determine the independent predictors among all factors related to CSS, univariate and multivariate Cox proportional hazard models were utilized for PMBNs patients with BM. Characteristics with *P* < 0.05 in the univariate Cox regression analysis were chosen for a further multivariate Cox regression analysis. The influence of variables on CSS was shown using hazard ratios (HRs) and corresponding 95% CIs.

Then, we constructed a nomogram based on the results of the multivariate Cox regression analysis. The prediction and discrimination performance of the nomogram were estimated using the bootstrap-corrected concordance index (*C*-index) and calibration curves. The value of the *C*-index, ranging from 0.5 to 1.0, indicated the result from random chance to perfect discrimination.

Additionally, receiver operating characteristic (ROC) curves were generated and the area under the curve (AUC) values were measured to assess the discriminative ability. A 3-fold calibration curve for 1- and 2-year CSS was graphically generated after 1,000 bootstrap resamples to estimate the concordance between the predicted survival and actual survival. Moreover, decision curve analysis (DCA) was performed to ascertain the clinical value of the nomogram.

All data analyses were performed using the SPSS 26.0 (IBM Corporation, Armonk, NY, USA) and R software version 3.5.0 (https://www.r-project.org/). Two-tailed *P* values less than 0.05 were considered statistically significant in all statistical analyses.

## 3. Results

### 3.1. Baseline Clinicopathological Features of Patients with or without BM

**Figure 2:**
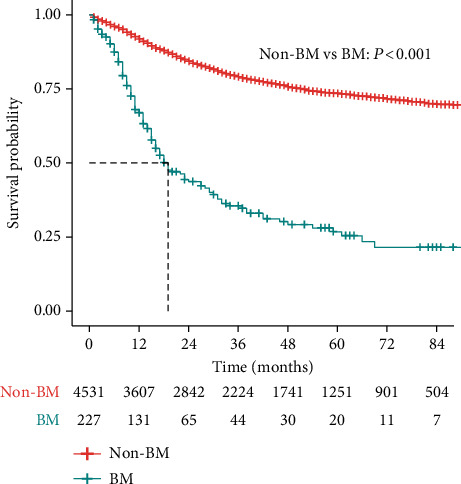
Kaplan-Meier curves of cancer-specific survival for PMBN patients with or without BM. The red line represented non-BM group, and the green line represented BM group. PMBN: primary malignant bone neoplasm; BM: bone metastasis.

The baseline clinicopathological features of the PMBNs patients with or without BM are shown in Table 1. Of the 4,758 patients in the analytic cohort, 227 (4.8%) patients had metastasis to bone at diagnosis. Significant differences were noted in clinical presentation between PMBNs patients with and without BM. The rate of BM among different types of bone tumors was significantly different (*P* < 0.05), with Ewing sarcoma having the highest rate (15.2%), followed by osteosarcoma (4.1%) and chondrosarcoma (1.6%), while chordoma had the lowest rate (0.7%). Patients with BM were younger than patients without BM (*P* = 0.001). Patients in the BM group were more likely to be unmarried and to have tumors of the axial sites, Ewing sarcoma, larger tumor size (>8 cm), and other distant metastatic diseases, with *P* values less than 0.05 for all comparisons. Compared to the non-BM group, patients in the BM group tended to undergo radiotherapy and chemotherapy rather than surgery. In contrast, the features of sex and race were comparable in both cohorts. As shown in Figure 2, the median CSS for patients with BM was 19 months. However, the median CSS in the non-BM group could not be calculated because the survival rate of patients without BM (79%) was over 50%. At the end of the follow-up, 137 patients with BM (60.4%) had died, and 133 of these deaths were related to cancer.

### 3.2. Risk Factors for Developing BM

Univariate logistic regression analyses were applied to analyze demographic and clinical characteristics, including age, sex, race, marital status, tumor site, tumor type, tumor grade, tumor size, tumor extension, and regional lymph node metastasis. After adjusting for potential confounding factors (shown as Table 2), the final results of multivariate logistic analyses indicated that axial primary site (OR = 1.770, 95% CI: 1.274-2.459, *P* = 0.001), other primary site (OR = 1.951, 95% CI: 1.163-3.275, *P* = 0.011), Ewing sarcoma (OR = 2.845, 95% CI: 1.941-4.170, *P* < 0.001), tumor extension beyond the periosteum (OR = 2.477, 95% CI: 1.567-3.917, *P* < 0.001), tumor diameter of 5-8 cm (OR = 3.403, 95% CI: 1.654-7.001, *P* = 0.001), tumor diameter over 8 cm (OR =5.562, 95% CI: 2.811-11.005, P <0.001), and regional lymph node metastasis (OR =2.900, 95% CI: 1.701-4.945, P <0.001) were associated with a higher risk of BM at initial diagnosis, while chondrosarcoma (OR = 0.439, 95% CI: 0.263-0.734, *P* = 0.002) and chordoma (OR = 0.117, 95% CI: 0.040-0.339, *P* < 0.001) were associated with a lower risk of BM than osteosarcoma.

### 3.3. Analysis of Survival and Prognostic Factors for Patients with BM

Of all patients, 227 BM-positive patients were included in the survival analysis. In the univariate analysis, age, marital status, tumor type, regional lymph node metastasis, liver metastasis, lung metastasis, surgery at the primary site, radiotherapy, chemotherapy, and the sequence of systemic therapy and surgery were significantly associated with CSS. In further multivariate analysis (Table 3), married status (HR = 2.457; 95% CI: 1.552-3.888; *P* < 0.001) and lung metastasis (HR = 1.934; 95% CI: 1.313-2.848; *P* = 0.001) were proven to be adverse independent prognostic factors. Compared with osteosarcoma, chondrosarcoma (HR = 0.342; 95% CI: 0.159-0.737; *P* = 0.006), Ewing sarcoma (HR = 0.592; 95% CI: 0.365-0.961; *P* = 0.034), and chordoma (HR = 0.015; 95% CI: 0.002-0.126; *P* < 0.001) had better prognoses. In contrast, surgery at the primary site (HR = 0.164; 95% CI: 0.073-0.369; *P* < 0.001) and chemotherapy (HR = 0.084; 95% CI: 0.037-0.192; *P* < 0.001) were positively correlated with better CSS, while radiotherapy (*P* = 0.175) had no significant effects.

### 3.4. Prognostic Nomogram for CSS for Patients with BM

**Figure 3:**
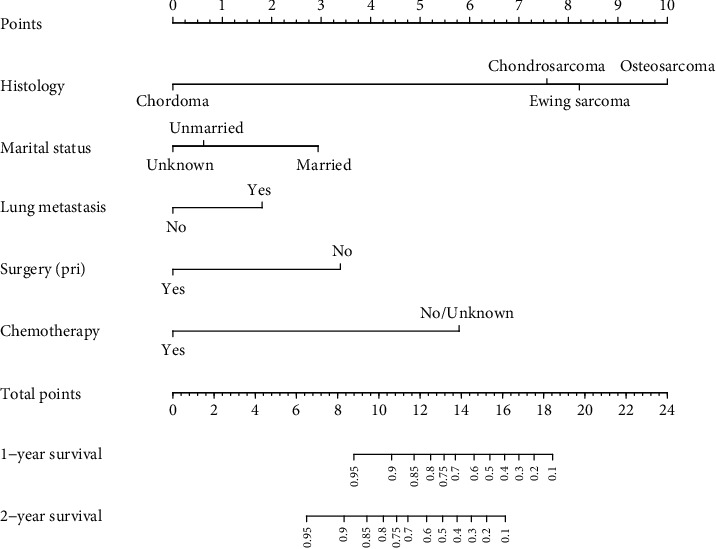
The nomogram of 1-year and 2-year cancer-specific survival for PMBN patients with BM. PMBN: primary malignant bone neoplasm; BM: bone metastasis.

On the basis of the above survival analysis, a nomogram model was constructed to predict 1-year and 2-year CSS for patients with BM. As shown in Figure 3, interestingly, pathological type had the greatest effect on prognosis, followed by chemotherapy and surgery at the primary site.

**Figure 4:**
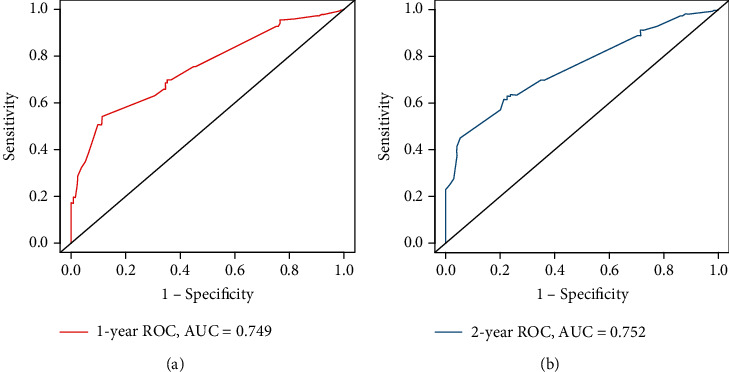
Receiver operating characteristic curve. (a) The area under ROC curve for the 1-year cancer-specific survival was 0.749; (b) The area under ROC curve for the 2-year cancer-specific survival was 0.752. ROC: receiver operating characteristic curve; AUC: area under curve.

**Figure 5:**
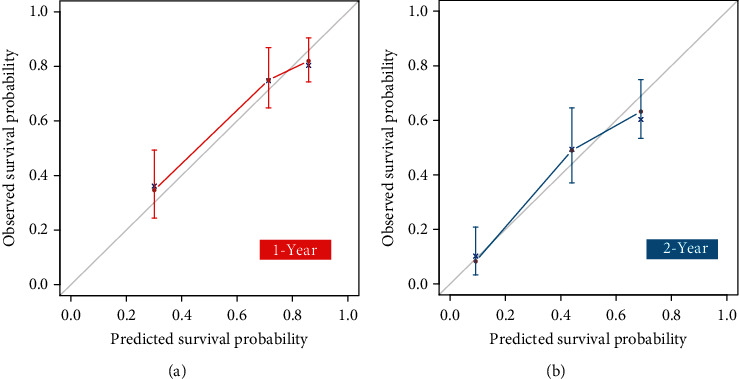
The calibration curves for predicting 1-year (a) and 2-year (b) cancer-specific survival of patients. The *x* axis represented the predicted probability of survival and the *y* axis represented the actual probability of survival. The 45° gray line indicated that the actuality agrees well with the prediction.

**Figure 6:**
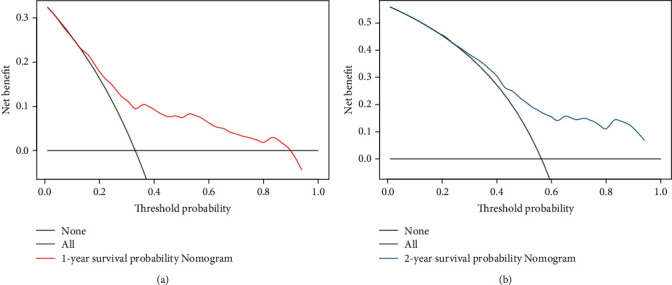
The decision curve analysis of the nomogram for the 1-year (a) and 2-year (b) cancer-specific survival probability. The *x* axis indicated the threshold probabilities, and the *y* axis measured the net benefit. The horizontal black line at *Y* = 0 assumed no patients died while the green line assumed that at a certain threshold probability all patients died. The red line (a) and the blue line (b) represented the net benefit of using the prognostic nomogram.

In our nomogram, the specific scores for every independent prognostic factor were obtained by drawing a straight line upward to the point axis. The scores of every variable were added to obtain the total score, and the predicted 1- and 2-year CSS was identified by finding a corresponding location on the survival axis. The bootstrap-corrected *C*-index was 0.733 (95% CI: 0.687-0.779). The AUC values for the 1-year and 2-year CSS were 0.749 and 0.752, respectively (Figure 4), indicating acceptable discrimination ability. In addition, calibration curves (Figure 5) showed that predicted survival was close to actual survival, demonstrating the ability of the nomogram to accurately predict 1-year and 2-year CSS. The decision curves (Figure 6) of the nomogram demonstrated good net benefit and clinical applicability in predicting patient survival.

## 4. Discussion

Due to the lack of sufficient clinical data on PMBNs patients with BM, studies conducted by single institutions are insufficient for investigating risk factors and prognostic factors. To our knowledge, this is the first population-based study focused on the BM of PMBNs patients at presentation. According to our findings, among the 4,758 patients with PMBNs, 227 (4.8%) patients presented with BM at initial diagnosis. Ewing sarcoma had the highest rate of BM (15.2%) among the four most common pathological types. We found that patients with tumors arising outside the extremities, Ewing sarcoma, tumors with larger size, tumors extending beyond the periosteum and metastasizing to regional lymph nodes were more predisposed to BM. In 227 patients with BM, univariate and multivariate Cox analysis revealed that married status and lung metastasis were associated with a poor prognosis and that osteosarcoma had a worse prognosis than the other three pathological subtypes. Nevertheless, surgery at the primary site and chemotherapy can help patients with BM obtain a better prognosis. Furthermore, we constructed a nomogram with excellent performance and clinical applicability to predict patient prognosis based on the results of multivariate survival analysis.

Our study pointed out that pathological type was an important factor influencing BM, and patients with Ewing sarcoma had the highest rate of developing BM, while patients with chordoma had the lowest rate. As a highly aggressive tumor, the rate of distant metastasis for Ewing sarcoma at first diagnosis has been reported to be approximately 20% [30, 31]. Chordoma was reported to be a low-grade, slow-growing, and locally invasive malignant tumor with a limited tendency to metastasize [32, 33]. The incidence of distant metastasis of chordoma in previous studies was approximately 7.7-8% [34, 35]. The tendency toward distant metastasis in different sarcomas, as reported previously, was consistent with our findings in this study.

In addition, bone tumors arising outside the extremities were more likely to have BM, which was also in agreement with other studies focused on distant metastasis [29, 36, 37]. On the one hand, we hypothesized that this finding might be related to the fact that the symptoms and signs of tumors in the axial bones usually appeared late and the tumor location was usually close to abdominal organs and large blood vessels, which increased the possibility of metastasis. [27, 38, 39] On the other hand, tumors in other atypical bones, such as the skull and mandible, might be more prone to metastasize to the bone than the limbs because of increased blood supply to these parts and the fact that radical surgery is almost impossible.

In addition, tumor size and extent of invasion are also significant risk factors for BM. As the period between tumor development and detection increases, the likelihood of tumor growth, extension, and metastasis increases. In addition, it is more difficult for surgeons to remove the tumor entirely and acquire proper margins when dealing with large tumors beyond the periosteum. Thus, the relationship between tumor size as well as extension and metastasis to the bone seemed rational. Although regional lymph node metastasis in bone malignancies is considered rare, with reported frequencies varying from <1% to 10% [25, 40–42], we found that regional node involvement plays an important role in BM. Thus, we recommend that doctors pay more attention to suspicious tumors and perform regional lymph node biopsy more aggressively in their clinical work. In addition, further researches are needed to elucidate the relationship between regional lymph node involvement and BM.

A whole-body 99Tc methylene diphosphonate (MDP) bone scan, a cost-effective and quick way to examine the entire skeleton, is commonly performed to assess bone metastases. A study claimed that in the detection of osteosarcoma, positron emission computed tomography (PET/CT) had better specificity, sensitivity, and overall accuracy than 99Tc MDP bone scans [43]. In addition, some scholars argue that whole-body magnetic resonance imaging (MRI), an alternative to a whole-body bone scan, also plays an important role in the detection of bone metastases. [44] Above all, paying attention to the aforementioned high-risk clinical characteristics in our study can help physicians identify patients with high BM risks as early as possible then carry out systemic examination and further treatment aggressively.

Furthermore, a prediction model presented in the form of a nomogram was established based on five independent prognostic factors we found in the survival analysis. In this model, the independent predictors for CSS were marital status, histological type, metastasis to the lung, surgery at the primary site, and chemotherapy, among which histological type had the most significant effect on prognosis. Patients with osteosarcoma have the worst prognosis, while patients with chordoma have the best prognosis (HR = 0.015; *P* < 0.001). This result was similar to that of a previous study on the survival of different osseous spinal tumors [45].

Interestingly, we found that marital status had an important effect on the prognosis of patients with BM. In recent years, marital status was thought to be an independent prognostic factor in prostate, breast, gastric, colorectal, and head/neck cancers [46–50]. In general, studies have declared that married patients have a better prognosis because of active health monitoring and better financial support between spouses [49, 50]. In contrast, unlike other studies, the results of our study suggested that married patients had a higher fatality rate. One reason we believed was that married patients tended to be older, and older patients with bone tumors might have a worse prognosis than adolescents [51–53]. Another reason we speculated was that married patients usually needed to bear more pressure from work, economy, and life, and the association between high psychological stress and poor prognosis of different types of cancers has been well documented [54, 55].

Pulmonary metastasis was found to be another factor contributing to a poor prognosis. Leo et al. identified multiple metastases at diagnosis (relative hazard ratio [RHR] = 2.3) as an independent risk factor for a poor prognosis. In his summary, only 8 (6.8%) of the 117 patients diagnosed with distant BM (isolated and combined with pulmonary metastasis) survived for more than 3 years. [56] We believe that when the patient has metastasis to both the bone and lung, the appropriate time for surgery has likely passed. Patients with multiple organ metastases are more likely to die from systemic multiple organ failures than those with single organ metastasis.

Surgery and chemotherapy were also shown to be factors that favored better survival among patients with BM. Currently, the standard treatment for malignant bone tumors includes surgery combined with chemotherapy [57, 58]. As the main treatment for malignant tumors, the effectiveness of surgery at the primary sites has been highlighted by an increasing number of recent studies, even for patients with distant metastasis [7, 59–61]. A study focused on osteosarcoma with primary metastasis reported that patients, even those with unresectable metastases, can still obtain better prognoses after surgical resection of the primary lesion [61]. The view that surgery of the primary lesion was beneficial for patients with BM was supported by this study. From our point of view, for patients who can tolerate surgery, the benefits of surgical resections of the primary sites were pain relief, prolongation of survival, and improvement in quality of life.

Bone sarcomas used to be considered incurable before the use of chemotherapy [62]. Arndt et al. argued that in patients with osteosarcoma or Ewing sarcoma, even localized disease would progress to distant metastasis and death without chemotherapy [63]. Cisplatin, methotrexate, ifosfamide, and doxorubicin are commonly used agents that have formed the basis of most chemotherapy regimental combinations over the past several decades [64–67]. Based on the above regimens, modern chemotherapy has achieved approximately 76% event-free survival at 3 years in osteosarcoma patients who have a good histological response [68]. Our study supports the advantages of chemotherapy in PMBNs patients with BM. In addition, our study found no improvement in the prognosis of patients with BM after radiotherapy, which is similar to the findings of other studies [12].

The clinical application of our nomogram can help clinicians accurately predict survival time and make informed clinical decisions. In addition, our predictive model could be helpful for further individual care and clinical trial design by integrating and unified analyzing various information. For patients who might have a poor prognosis according to the model, more physiological and psychological support as well as more palliative treatments may be needed. Active participation in clinical trials of anticancer drugs may also present a chance to prolong survival.

Inevitably, our study had several potential limitations. First, as a retrospective study, missing data and selection bias were unavoidable in the present research. Second, since asymptomatic patients and subsequent bone metastases during disease progression were not recorded in the SEER database, the incidence we obtained in this study might be lower than the actual rate. Third, no specific details about surgery and chemotherapy were included in the SEER database, such as surgical approaches, chemotherapy regimens, and drug dosage. Hence, we were unable to recommend an accurate treatment plan for patients. Fourth, because of the rarity of BMs, we did not have sufficient data from other sources. Although self-sampling internal verification confirms the good predictive performance of the nomogram, formal external verification is still needed. With the increase in new clinical trial data, our model should be further enhanced and verified.

## 5. Conclusion

In conclusion, the present study based on the SEER database provides a deep understanding of PMBNs with BM. Our study proved that risk factors correlated with BM in PMBNs patients include Ewing sarcoma, tumor size over 5 cm, tumors arising outside the extremities, tumors extending beyond the periosteum, and the presence of regional lymph node involvement. These risk factors could potentially be used in clinical surveillance to improve the early detection of BM in PMBNs patients. Furthermore, a nomogram to predict the CSS of BM in patients with PMBNs was constructed based on five independent prognostic factors, including pathological type, marital status, pulmonary metastasis, surgery, and chemotherapy. We believe that this nomogram can assist in accurate prognostic prediction and individual clinical decision-making.

## Figures and Tables

**Table 1:** Baseline of the demographic and related clinical characteristics of enrolled patients with and without bone metastasis (BM).

	Non-BM *n* = 4,531	BM *n* = 227	Total *n* = 4,758	*P* value
Age, years				0.001
<18	1,052 (23.2%)	76 (33.5%)	1,128 (23.7%)	
18—64	2,802 (61.9%)	129 (56.8%)	2,931 (61.6%)	
≥65	677 (14.9%)	22 (9.7%)	699 (14.7%)	
Sex				0.115
Female	2,005 (44.3%)	88 (38.8%)	2,093 (44.0%)	
Male	2,526 (55.7%)	139 (61.2%)	2,665 (56.0%)	
Race				0.712
White	3,641 (80.4%)	179 (78.9%)	3,820 (80.3%)	
Black	439 (9.7%)	22 (9.7%)	461 (9.7%)	
Other	408 (9.0%)	25 (11.0%)	433 (9.1%)	
Unknown	43 (0.9%)	1 (0.4%)	44 (0.9%)	
Marital status				<0.001
Unmarried	2,818 (62.2%)	173 (76.2%)	2,991 (62.8%)	
Married	1,530 (33.8%)	48 (21.2%)	1,578 (33.2%)	
Unknown	183 (4.0%)	6 (2.6%)	189 (4.0%)	
Primary site				<0.001
Extremities	2,445 (54.0%)	95 (41.9%)	2,540 (53.4%)	
Axial	1,418 (31.3%)	107 (47.1%)	1,525 (32.0%)	
Others	668 (14.7%)	25 (11.0%)	693 (14.6%)	
Histology type				<0.001
Osteosarcoma	1,779 (39.3%)	77 (33.9%)	1,856 (39.0%)	
Chondrosarcoma	1,510 (33.3%)	24 (10.6%)	1,534 (32.2%)	
Ewing sarcoma	680 (15.0%)	122 (53.7%)	802 (16.9%)	
Chordoma	562 (12.4%)	4 (1.8%)	566 (11.9%)	
Grade				<0.001
Low	578 (12.8%)	3 (1.3%)	581(12.2%)	
High	2,343 (51.7%)	91 (40.1%)	2,434 (51.2%)	
Unknown	1,610 (35.5%)	133 (58.6%)	1,743 (36.6%)	
Extension				<0.001
Inside periosteum	1,486 (32.8%)	26 (11.5%)	1,512 (31.8%)	
Beyond periosteum	1,647 (36.3%)	116 (51.1%)	1,763 (37.1%)	
Unknown	1,398 (30.9%)	85 (37.4%)	1,483 (31.1%)	
Tumor size, cm				<0.001
≤5	1,177 (26.0%)	10 (4.4%)	1,187 (24.9%)	
5-8	990 (21.8%)	37 (16.3%)	1,027 (21.6%)	
>8	1,779 (39.3%)	117 (51.5%)	1,896 (39.9%)	
Unknown	585 (12.9%)	63 (27.8%)	648 (13.6%)	
RLNs metastasis				<0.001
No	3,272 (72.2%)	132 (58.1%)	3,404 (71.6%)	
Yes	92 (2.0%)	24 (10.6%)	116 (2.4%)	
Unknown	1,167 (25.8%)	71 (31.3%)	1,238 (26.0%)	
Brain metastasis				<0.001
No	4,521 (99.8%)	219 (96.5%)	4,740 (99.6%)	
Yes	10 (0.2%)	8 (3.5%)	18 (0.4%)	
Liver metastasis				<0.001
No	4,517 (99.7%)	212 (93.4%)	4,729 (99.4%)	
Yes	14 (0.3%)	15 (6.6%)	29 (0.6%)	
Lung metastasis				<0.001
No	4,036 (89.1%)	124 (54.6%)	4,160 (87.4%)	
Yes	495 (10.9%)	103 (45.4%)	598 (12.6%)	
Surgery (pri)				<0.001
No	671 (14.8%)	152 (67.0%)	823 (17.3%)	
Yes	3,860 (85.2%)	75 (33.0%)	3,935 (82.7%)	
Surgery (dis)				0.022
No	4,343 (95.9%)	209 (92.1%)	4,552 (95.7%)	
Yes	180 (4.0%)	17 (7.5%)	197 (4.1%)	
Unknown	8 (0.2%)	1 (0.4%)	9 (0.2%)	
Radiotherapy				<0.001
No/unknown	3,518 (77.6%)	114 (50.2%)	3,632 (76.3%)	
Yes	1,013 (22.4%)	113 (49.8%)	1,126 (23.7%)	
Chemotherapy				<0.001
No/unknown	2,236 (49.3%)	30 (13.2%)	2,266 (47.6%)	
Yes	2,295 (50.7%)	197 (86.8%)	2,492 (52.4%)	
Sequence				0.001
No	2,614 (57.7%)	160 (70.5%)	2,774 (58.3%)	
Before surgery	555 (12.2%)	15 (6.6%)	570 (12.0%)	
After surgery	420 (9.3%)	20 (8.8%)	440 (9.2%)	
Others	942 (20.8%)	32 (14.1%)	974 (20.5%)	

BM: bone metastasis; RLNs: regional lymph nodes; Surgery (pri): surgery of primary site; Surgery (dis): surgery of distant metastasis; Sequence: the sequence of systemic therapy and surgery.

**Table 2:** Univariate and multivariate logistic regression analysis for risk factors of bone metastasis (BM).

Variables	Univariate analysis		Multivariate analysis	
	OR (95% CI)	*P*	OR (95% CI)	*P*
Age, years				
<18	Reference		Reference	
18—64	0.637 (0.476-0.853)	0.003	1.086 (0.776-1.521)	0.630
≥65	0.450 (0.277-0.730)	0.001	1.378 (0.766-2.482)	0.285
Sex				
Female	Reference			
Male	1.254 (0.954-1.648)	0.105		
Race				
White	Reference			
Black	1.109 (0.648-1.605)	0.934		
Other	1.246 (0.810-1.918)	0.316		
Unknown	NA			
Marital status				
Unmarried	Reference		Reference	
Married	0.511 (0.369-0.708)	<0.001	1.091 (0.735-1.621)	0.666
Unknown	NA		NA	
Primary site				
Extremities	Reference		Reference	
Axial	1.942 (1.462-2.580)	<0.001	1.770 (1.274-2.459)	0.001
Others	0.963 (0.615-1.509)	0.870	1.951 (1.163-3.275)	0.011
Histology type				
Osteosarcoma	Reference		Reference	
Chondrosarcoma	0.367 (0.231-0.584)	<0.001	0.439 (0.263-0.734)	0.002
Ewing sarcoma	4.145 (3.075-5.588)	<0.001	2.845 (1.941-4.170)	<0.001
Chordoma	0.164 (0.060-0.451)	<0.001	0.117 (0.040-0.339)	<0.001
Grade				
Low	Reference		Reference	
High	7.483 (2.361-23.720)	0.001	3.017 (0.919-9.904)	0.069
Unknown	NA		NA	
Extension				
Inside periosteum	Reference		Reference	
Beyond periosteum	4.025 (2.616-6.194)	<0.001	2.477 (1.567-3.917)	<0.001
Unknown	NA		NA	
Tumor size, cm				
≤5	Reference		Reference	
5-8	4.399 (2.176-8.891)	<0.001	3.403 (1.654-7.001)	0.001
>8	7.741 (4.041-14.827)	<0.001	5.562 (2.811-11.005)	<0.001
Unknown	NA		NA	
RLNs metastasis				
No	Reference		Reference	
Yes	6.466 (3.994-10.469)	<0.001	2.900 (1.701-4.945)	<0.001
Unknown	NA		NA	

OR: odds ratio; CI: confidence interval; NA: not applicable; RLNs: regional lymph nodes.

**Table 3:** Univariate and multivariate cox regression analysis for prognostic factors of patients with bone metastasis (BM).

Variables	Univariate analysis		Multivariate analysis	
	HR (95% CI)	*P*	HR (95% CI)	*P*
Age, years				
<18	Reference		Reference	
18—64	1.693 (1.144-2.505)	0.008	1.177 (0.742-1.867)	0.489
≥65	3.232 (1.793-5.827)	<0.001	2.010 (0.858-4.711)	0.108
Sex				
Female	Reference			
Male	0.740 (0.525-1.043)	0.086		
Race				
White	Reference			
Black	1.375 (0.824-2.295)	0.223		
Other	0.517 (0.262-1.022)	0.058		
Unknown	NA			
Marital status				
Unmarried	Reference		Reference	
Married	2.762 (1.865-4.093)	<0.001	2.457 (1.552-3.888)	<0.001
Unknown	2.792 (1.127-6.918)	0.027	0.836 (0.273-2.563)	0.754
Primary site				
Extremities	Reference			
Axial	0.888 (0.619-1.272)	0.516		
Others	0.999 (0.557-1.791)	0.997		
Histology type				
Osteosarcoma	Reference		Reference	
Chondrosarcoma	1.230 (0.69-2.192)	0.484	0.342 (0.159-0.737)	0.06
Ewing sarcoma	0.647 (0.448-0.935)	0.02	0.592 (0.365-0.961)	0.034
Chordoma	0.304 (0.042-2.209)	0.24	0.015 (0.002-0.126)	<0.001
Grade				
Low	Reference			
High	2.127 (0.293-15.446)	0.456		
Unknown	NA			
Extension				
Inside periosteum	Reference			
Beyond periosteum	0.970 (0.563-1.671)	0.912		
Unknown	NA			
Tumor size, cm				
≤5	Reference			
5-8	0.722 (0.288-1.811)	0.488		
>8	0.678 (0.293-1.567)	0.363		
Unknown	NA			
RLNs metastasis				
No	Reference		Reference	
Yes	1.956 (1.184-3.234)	0.009	1.471 (0.852-2.542)	0.166
Unknown	NA		NA	
Brain metastasis				
No	Reference			
Yes	0.844 (0.312-2.284)	0.738		
Liver metastasis				
No	Reference		Reference	
Yes	2.654 (1.423-4.949)	0.002	1.248 (0.623-2.503)	0.532
Lung metastasis				
No	Reference		Reference	
Yes	1.810(1.284-2.552)	0.001	1.934 (1.313-2.848)	0.001
Surgery (pri)				
No	Reference		Reference	
Yes	0.572 (0.383-0.856)	0.007	0.164 (0.073-0.369)	<0.001
Surgery (dis)				
No	Reference			
Yes	0.831 (0.387-1.784)	0.635		
Unknown	NA			
Radiotherapy				
No/unknown	Reference		Reference	
Yes	0.659 (0.468-0.928)	0.017	0.761 (0.513-1.129)	0.175
Chemotherapy				
No/unknown	Reference		Reference	
Yes	0.317 (0.201-0.499)	<0.001	0.084 (0.037-0.192)	<0.001
Sequence				
No	Reference		Reference	
Before surgery	0.551 (0.256-1.186)	0.128	2.700 (0.945-7.720)	0.064
After surgery	0.472 (0.220-1.104)	0.054	1.885 (0.751-4.734)	0.177
Others	0.435 (0.234-0.810)	0.009	2.257 (0.825-6.174)	0.113

HR: hazard ratio; CI: confidence interval; NA: not applicable; RLNs: regional lymph nodes; Surgery (pri): surgery of primary site; Surgery (dis): surgery of distant metastasis; Sequence: the sequence of systemic therapy and surgery.

## Data Availability

The publicly available datasets used and/or analyzed during the current study are available in the Surveillance, Epidemiology, and End Results (SEER) Database (https://seer.cancer.gov/.).

## Abbreviations

PMBNs: Primary malignant bone neoplasms
BM: Bone metastasis
SEER: the Surveillance, Epidemiology, and End Results database
AJCC: the American Joint Committee on Cancer
ICD-O: the International Classification of Diseases for Oncology OR odds ratio
HR: Hazard ratio
CI: Confidence interval
MDP: Methylene diphosphonate
PET/CT: Positron emission computed tomography
MRI: Magnetic resonance imaging.
